# Non-English Preferred Language and Hospital Mortality in Candidemia: A Retrospective Cohort Study

**DOI:** 10.1093/ofid/ofag626

**Published:** 2026-10-08

**Authors:** Hamail Iqbal, Ahmet Dalkilic, Dejan Nikolic, Carlo Foppiano Palacios

**Affiliations:** Cooper Medical School of Rowan University, Camden, New Jersey, USA; Cooper Medical School of Rowan University, Camden, New Jersey, USA; Cooper Medical School of Rowan University, Camden, New Jersey, USA; Department of Pathology, Cooper University Hospital, Camden, New Jersey, USA; Cooper Medical School of Rowan University, Camden, New Jersey, USA; Division of Infectious Diseases, Cooper University Hospital, Camden, New Jersey, USA

**Keywords:** candidemia, mortality, outcomes, social determinants of health

## Abstract

We evaluated associations between social determinants of health and mortality in candidemia via a retrospective cohort (2017–2022). Among 237 episodes, non-English language was independently associated with higher mortality (aOR 3.22, 95% CI 1.03–10.08). Other social determinants were not significant. Non-English preferred language may identify an important difference in candidemia outcomes which warrants further investigation and validation in larger, multicenter studies.

Candidemia is the most common manifestation of invasive candidiasis and remains an important cause of hospital-acquired bloodstream infections with recent surveillance estimates of 25 000 cases annually in the United States [1]. Despite advances in antifungal therapies, outcomes remain poor. Crude in-hospital mortality approaches 25%, and 30-day mortality rates may reach up to 40% [2].

Although traditional risk factors for candidemia are well established, there is increasing recognition that social and structural determinants influence both exposure risk and clinical outcomes. Surveillance data reveal considerable disparities in candidemia incidence, with higher rates observed among Black patients, and population-level studies indicate elevated rates of invasive fungal infections in lower-income communities [3]. Additionally, shifts in substance use epidemiology have added to rising candidemia incidence among younger adults [4]. However, few studies have examined whether patient-level social determinants of health (SDOHs), particularly a preferred language other than English, are associated with mortality among hospitalized patients with candidemia, or whether differences in care processes contribute to outcome disparities [2].

This study sought to evaluate the association between SDOHs, particularly non-English preferred language, and mortality among patients with candidemia. Our findings may inform future interventions to improve care for vulnerable populations.

## METHODS

### Study Design and Population

A retrospective cohort study was conducted at a tertiary academic medical center in Camden, NJ. All patients ≥18 years with at least 1 blood culture positive for *Candida* species (including species reclassified) from January 1, 2017 to December 31, 2022, were included. Candidemia cases were excluded if infection occurred before age 18 or if death occurred before blood culture flagged positive for yeast or antifungal treatment was initiated. Multiple candidemia episodes per patient were allowed. However, repeat hospitalizations involving the same *Candida* species were treated as a single episode if the patient was hospitalized prior to completing antifungal therapy prescribed at the previous discharge. This study-specific episode definition was based on clinical treatment course and Candida species rather than the CDC surveillance definition, which uses a 30-day interval from the index positive culture to distinguish recurrent cases. The study protocol was approved by the Cooper University Healthcare Institutional Review Board (IRB number 23-007).

### Data Collection

Patient-level demographic and clinical data of candidemia episodes were recorded from the electronic health record (EHR). Variables included age, sex, diabetes, end-stage renal disease (ESRD), presence of a central venous catheter, receipt of total parenteral nutrition, recent gastrointestinal surgery or trauma, shock requiring vasopressor support, respiratory failure requiring mechanical ventilation, and fungal species identification (*Candida albicans* vs non-*albicans*). Candidemia management process measures included repeat blood cultures and a formal ophthalmologic examination [5].

### Social Determinants Collection

Patient-level SDOH variables obtained from EHR documentation included non-English preferred language, housing insecurity, insurance status, history of substance use, and partnered marital status. Preferred language was routinely collected during the admission intake process and documented in a structured EHR field. There were no missing preferred-language data in the study cohort but we note that preferred language is an imperfect proxy for limited English proficiency and does not capture interpreter use. Insurance was classified for each patient into mutually exclusive categories: private only, Medicare only, Medicaid only, dual coverage, or uninsured. Neighborhood socioeconomic disadvantage was assessed using the national area deprivation index (ADI) percentile, assigned at the census tract level based on the patient's residential address documented in the EHR. ADI values were derived from the Neighborhood Atlas (Center for Health Disparities Research, University of Wisconsin School of Medicine and Public Health), with higher a percentile reflecting greater deprivation [6, 7].

### Outcomes and Statistical Analysis

The primary outcome was hospital mortality, defined as death during hospitalization or discharge to hospice. Counts with percentages were used for categorical variables, while medians (IQRs) were used for continuous variables due to non-normal distributions. Bivariate associations between categorical exposures (SDOH variables, race, and Latino ethnicity) and hospital mortality were evaluated using Fisher's exact tests. Comparisons of national ADI percentiles were performed using the Wilcoxon rank-sum test, due to non-normal distributions.

Multivariable logistic regression estimated adjusted odds ratios (aORs) with 95% confidence intervals (CIs). Covariates were prespecified based on clinical relevance rather than stepwise selection. Given the limited number of outcome events, model complexity was restricted to reduce the risk of overfitting and unstable effect estimates. Thus, not all potential candidemia risk factors were included as covariates. The primary model (Model A) evaluated non-English language preference and mortality, with secondary analyses assessing other SDOH, race/ethnicity and neighborhood deprivation using analogous models (Supplementary Supplement). Separate models were used for individual SDOH exposures to improve sensitivity given the limited number of outcome events. Model B additionally incorporated candidemia care-process variables as an exploratory analysis to assess whether the association between preferred language and mortality persisted after accounting for measured differences in care processes. In post hoc sensitivity analyses, we assessed the potential impact of within-patient correlation from recurrent candidemia episodes using patient-clustered robust standard errors and separately restricting the analysis to the first candidemia episode per patient. Data were complete for the primary outcome, preferred language, insurance status, substance use history, partnered status, race/ethnicity, and all clinical covariates included in the multivariable models. Housing insecurity was missing for 35 episodes (14.8%) and ADI for 10 episodes (4.2%), models evaluating these exposures were performed using complete cases. All statistical analyses were performed using R version 4.0.2, with statistical significance defined as *P* < .05.

## RESULTS

### Demographics and Baseline Characteristics

We included 199 patients, accounting for 237 episodes of candidemia. The median age was 55.0 years (IQR 37–68.5), and 55.3% (N = 110/199) of patients were male. Most patients were White (57.8%, N = 115/199) or Black (22.6%, N = 45/199); 15.1% (N = 30/199) identified as Latino. The most common risk factor was gastrointestinal surgery or trauma in 35.2% (N = 70/199) of patients.

### Social Determinants of Health

Among 199 patients with candidemia, 34.7% (N = 69/199) reported a history of substance use, 33.2% (N = 66/199) were partnered, 30.7% (N = 61/199) had documented housing insecurity, and 9.5% (N = 19/199) indicated a non-English preferred language. Patients reporting a non-English preferred language were older than English-preferring patients (median age 67.0 [IQR 57.5–74.0] vs 49.0 [34.0–65.0] years; *P* = .002). Insurance providers were Medicare only (31.7%, N = 63/199), Medicaid only (27.6%, N = 55/199), privately insured (22.1%, N = 44/199), dual Medicare–Medicaid (13.6%, N = 27/199), or uninsured (5.0%, N = 10/199). Median national ADI percentile was 55.0 (IQR 42.0–77.8).

### Clinical Severity, Microbiology, and Care-process Measures

Across the 237 candidemia episodes (Table 1), 35.9% (N = 85) involved vasopressor support and 29.1% (N = 69) required mechanical ventilation. *Candida albicans* was identified in 38.0% (N = 90/237) of episodes. Repeat blood cultures were obtained in 96.6% (N = 229/237) of episodes, and ophthalmologic examinations were performed in 81.9% (N = 194/237).

**Table 1.** Demographic, Social, and Clinical Characteristics of Candidemia Episodes, Stratified by Hospital Mortality

Characteristic	Overall (N = 237)	No Hospital Mortality (N = 183)	Hospital Mortality (N = 54)	*P* Value
Demographic characteristics				
Age, median (IQR), y	50.0 (35.0–67.0)	44.0 (32.5–63.5)	63.5 (53.5–71.0)	<.01
Male sex	136 (57.4)	108 (59.0)	28 (51.9)	.353
Race				.450
White	128 (54.0)	103 (56.3)	25 (46.3)	
Black	69 (29.1)	52 (28.4)	17 (31.5)	
Asian	5 (2.1)	4 (2.2)	1 (1.9)	
Other	35 (14.8)	24 (13.1)	11 (20.4)	
Hispanic or Latino ethnicity	31 (13.1)	22 (12.0)	9 (16.7)	.366
Social determinants of health				
Non-English preferred language	19 (8.0)	9 (4.9)	10 (18.5)	.003
Housing insecurity^a^	66 (32.7)	60 (37.7)	6 (14.0)	.003
Insurance category				.040
Private only	46 (19.4)	34 (18.6)	12 (22.2)	
Dual coverage	33 (13.9)	25 (13.7)	8 (14.8)	
Medicaid only	77 (32.5)	68 (37.2)	9 (16.7)	
Medicare only	68 (28.7)	46 (25.1)	22 (40.7)	
Uninsured/none	13 (5.5)	10 (5.5)	3 (5.6)	
Any history of substance use	74 (31.2)	66 (36.1)	8 (14.8)	.003
Partnered marital status	75 (31.6)	55 (30.1)	20 (37.0)	.405
ADI, median (IQR)^a^	55.0 (42.0–82.5)	55.0 (41.8–85.0)	55.0 (43.5–69.0)	.981
Clinical characteristics				
Diabetes	65 (27.4)	47 (25.7)	18 (33.3)	.299
End-stage renal disease	9 (3.8)	6 (3.3)	3 (5.6)	.430
Central venous catheter^b^	3 (1.3)	3 (1.6)	0 (0)	1.000
Total parenteral nutrition	53 (22.4)	46 (25.1)	7 (13.0)	.065
Gastrointestinal surgery or trauma	100 (42.2)	79 (43.2)	21 (38.9)	.639
Vasopressor required	85 (35.9)	46 (25.1)	39 (72.2)	<.001
Mechanical ventilation	69 (29.1)	37 (20.2)	32 (59.3)	<.001
*Candida albicans*	90 (38.0)	75 (41.0)	15 (27.8)	.082
Care-process measures				
Repeat blood cultures obtained	229 (96.6)	182 (99.5)	47 (87.0)	<.001
Ophthalmologic examination	194 (81.9)	159 (86.9)	35 (64.8)	.001

Data are n (%) unless otherwise indicated. *P* values were calculated using Fisher's exact test for categorical variables and the Wilcoxon rank-sum test for continuous variables.

^a^Housing insecurity was available for 202 episodes and ADI for 227 episodes; percentages are calculated among episodes with available data.

^b^Central venous catheter presence was ascertained from structured electronic health record documentation. The observed proportion is substantially lower than expected in a candidemic cohort and likely reflects incomplete capture in the fields available for retrospective abstraction rather than true prevalence; this variable was therefore reported descriptively and not included in multivariable models.

### Hospital Mortality

Hospital mortality occurred in 54 of 237 episodes (27.1%). Bivariate analysis (Table 1) revealed that non-English preferred language was significantly more prevalent among episodes with mortality than among survivors (18.5% vs 4.9%, *P* = .003). In contrast, housing insecurity and a history of substance use were less frequent among mortality cases (14.0% vs 37.7%, *P* = .003; and 14.8% vs 36.1%, *P* = .003, respectively). Insurance status also varied by outcome (*P* = .040), with higher rates of Medicare-only coverage (40.7% vs 25.1%) and lower Medicaid-only coverage (16.7% vs 37.2%) among mortality cases. There was no significant association between hospital mortality and race (*P* = .450), Hispanic/Latino ethnicity (*P* = .366), partnered status (*P* = .405), or national ADI percentile (median 55 in both groups; *P* = .981) in unadjusted analyses.

In the primary multivariable logistic regression analysis (Model A; N = 237 episodes, Table 2), non-English preferred language remained independently associated with increased odds of hospital mortality (aOR 3.22, 95% CI 1.03–10.08). Advancing age (aOR 1.04 per year, 95% CI 1.02–1.07) and the need for vasopressor support (aOR 4.74, 95% CI 1.67–13.58) were also independently associated with higher mortality risk. Sex, diabetes, ESRD, mechanical ventilation, and *C. albicans* (vs non-albicans) were not significantly associated with mortality after adjustment (all 95% CIs included 1.0). In sensitivity analyses accounting for repeated observations, the magnitude of the association between non-English preferred language and hospital mortality was similar when using patient-clustered robust standard errors (aOR, 3.22; 95% CI, .97–10.69) and when restricting the analysis to the first candidemia episode per patient (N = 199; aOR, 3.18; 95% CI, 1.01–10.11) (Supplementary Table 1).

**Table 2.** Multivariable Logistic Regression Models for Hospital Mortality (in-hospital Death or Discharge to Hospice) Among Adults With Candidemia: Model A (Primary) and Model B (Exploratory Care-process–Adjusted)

	Model A^a^		Model B^a,b^	
Predictor	aOR (95% CI)	*P*	aOR (95% CI)	*P*
Non-English preferred language	3.22 (1.03–10.08)	.042	4.97 (1.45–17.33)	.011
Age (per year)	1.04 (1.02–1.07)	<.001	1.04 (1.02–1.07)	.002
Male sex	0.66 (0.31–1.37)	.266	0.67 (0.30–1.47)	.319
Diabetes	0.82 (0.36–1.82)	.629	0.74 (0.30–1.78)	.512
ESRD	0.74 (0.11–4.25)	.745	0.78 (0.10–5.24)	.798
Vasopressor required	4.74 (1.67–13.58)	.003	3.97 (1.26–12.68)	.018
Mechanical ventilation	1.70 (0.61–4.80)	.310	2.86 (0.90–9.70)	.081
*Candida albicans* (vs non-*albicans*)	0.70 (0.32–1.50)	.364	0.74 (0.31–1.71)	.490
Repeat blood cultures obtained			0.05 (0.002–0.41)	.015
Ophthalmologic exam completed			0.21 (0.07–0.57)	.002

^a^Both models included 237 candidemia episodes.

^b^Model B additionally adjusts for candidemia care-process variables (repeat blood cultures and ophthalmologic exam) and is presented as an exploratory analysis.

In exploratory swap-in models (A2-A5), housing insecurity, insurance status, history of substance use, or marital status were not independently associated with hospital mortality even after adjustment for age and illness severity (Supplementary Tables 2–5). Notably non-English preferred language remained significantly associated with mortality even after adjustment for race and ethnicity (A-Race: aOR 4.56, 95% CI 1.19–18.81; *P* = .029), while Black race (aOR 1.40, 95% CI 0.59–3.28; *P* = .440) and Hispanic/Latino ethnicity (aOR 0.63, 95% CI 0.16–2.12; *P* = .473) were not (Supplementary Table 6). The addition of national ADI percentile to the primary model also demonstrated no independent association (Supplementary Table 7) with mortality (ADI aOR 1.00, 95% CI 0.98–1.02; *P* = .916), and the association between non-English preferred language and mortality persisted (AN: aOR 3.98, 95% CI 1.22–13.39; *P* = .022). In Model B (N = 237), the association between non-English preferred language and hospital mortality remained significant (aOR 4.97, 95% CI 1.45–17.33). Obtaining repeat blood cultures (aOR 0.05, 95% CI 0.002–0.41) and the completion of ophthalmologic examination (aOR 0.21, 95% CI 0.07–0.57) were independently associated with lower odds of mortality after adjustment.

## DISCUSSION

In our primary adjusted analysis, patients with a non-English preferred language had significantly higher odds of hospital mortality (Model A aOR 3.22, 95% CI 1.03–10.08). This association persisted after additional adjustment for candidemia care-process measures (Model B aOR 4.97, 95% CI 1.45–17.33). To our knowledge, this is the first study to specifically examine language-related disparities in candidemia outcomes, highlighting the need to further evaluate communication-related barriers and interventions for patients who prefer a non-English language, including timely access to professional interpreter services and linguistically appropriate communication in this high-mortality infection.

In our cohort, non-English preferred language was an independent predictor of hospital mortality. Although patients reporting a non-English language were older, the association between non-English preferred language and hospital mortality persisted after multivariable adjustment. The persistence of this association suggests that language barriers may introduce challenges that are not fully captured by process measures alone. For instance, repeat blood cultures and ophthalmologic examinations, which were associated with better outcomes, could be less commonly performed in patients who deteriorate quickly or transition to comfort care, introducing potential survivorship bias. The management of candidemia is time-sensitive and requires coordination across multiple specialties, making effective communication critical for optimal care [8, 9]. Potential mechanisms underlying the observed association may include delayed symptom reporting, difficulties navigating complex decisions such as source control, and challenges with diagnostic workup for metastatic complications [10]. Language barriers can also impede transitions of care, affecting access and adherence to antifungals, understanding discharge instructions, and completing necessary follow-up [11]. Although language-related disparities in candidemia have not been widely studied, our findings align with broader literature that language barriers are linked to increased risk of medical errors and worse clinical outcomes, underscoring the importance of language-concordant care and professional interpreter services in candidemia management [12].

In secondary analyses, we did not observe independent associations between hospital mortality and other SDOH variables, including housing insecurity, insurance status, history of substance use, partnered status, race, ethnicity, or neighborhood deprivation (national ADI percentile). Notably, diabetes and ESRD, both established risk factors for developing candidemia, were not associated with hospital mortality in either bivariate or multivariable analyses, consistent with the distinction between factors that predispose to candidemia and those associated with death once infection occurs. Interestingly, housing insecurity, substance use history, and Medicaid coverage appeared inversely associated with mortality in unadjusted analyses, likely due to confounding by age and illness severity. Our null findings should be interpreted with caution given the modest number of mortality events (N = 54) and the use of area-level proxies, which may not reflect individual socioeconomic circumstances. Notably, large population-based studies have consistently demonstrated persistent racial disparities in mortality across multiple infectious syndromes, indicating that our findings do not rule out the presence of structural inequities [13, 14]. Previous work on SDOH and candidemia has primarily focused on infection risk rather than outcomes, including a Colorado study which found a 15% higher candidemia incidence per 0.1-unit increase in social vulnerability index [15]. These observations underscore the need for larger multicenter studies to better define the influences of SDOH on both candidemia acquisition and clinical outcomes.

This study has several limitations. First, the retrospective single-center design limits generalizability and leaves the potential for residual confounding despite prespecified adjustment. Second, clinical and SDOH variables were abstracted from EHR documentation and are subject to incomplete ascertainment and misclassification. Central venous catheter presence, for example, was documented in only 1.3% of episodes and could not be reliably characterized or modeled. Housing insecurity and ADI were missing for 14.8% and 4.2% of episodes, respectively, and analyses of these exposures were restricted to complete cases, introducing the potential for selection bias. Lack of documentation of housing insecurity or substance use, for example, may not reliably indicate absence of these exposures. In particular, non-English preferred language is an imperfect proxy for limited English proficiency and does not capture interpreter use or communication quality. At the time of this study, record of interpreter use during clinical encounters was difficult to reliably extract from the EHR. Third, care-process variables in Model B may mediate the relationship between language preference and mortality and are prone to survivorship bias, since early deaths reduce the likelihood of receiving these interventions. Fourth, our study-specific definition of recurrent candidemia episodes differed from the CDC surveillance definition, potentially resulting in episode misclassification. Because patients could contribute multiple episodes, we conducted sensitivity analyses using patient-clustered robust standard errors and restricting the cohort to the first episode per patient: the point estimate was stable across both approaches, although the association was attenuated to borderline significance when accounting for within-patient correlation. Fifth, the modest number of mortality events limited power for subgroup and interaction testing. Sixth, our composite hospital mortality outcome included both in-hospital death and discharge to hospice. Therefore, patients discharged to hospice who subsequently survived may have been misclassified as having experienced the mortality outcome. Postdischarge vital status was not independently verified, and future studies incorporating linkage to death records could provide more precise mortality ascertainment. Finally, the relatively small number of patients with a non-English preferred language limited statistical precision, as reflected by the wide CIs, and these findings require validation in larger, linguistically diverse, multicenter cohorts.

## CONCLUSION

Non-English preferred language was associated with higher mortality among adults with candidemia. In contrast, race, ethnicity, insurance status, housing insecurity, substance use history, partnered status, and neighborhood deprivation (national ADI) were not significantly associated with mortality, although limited power and sparse subgroups may have limited our ability to detect smaller associations. Multicenter studies with greater linguistic diversity are needed to validate these findings and mechanisms underlying the observed association, including communication barriers, interpreter use, and differences in care delivery. Understanding these mechanisms may identify and evaluate targeted interventions to address language-related disparities in candidemia outcomes.

## Supplementary Material

ofag626_Supplementary_Data

## Supplementary Data

Supplementary materials are available at *Open Forum Infectious Diseases* online. Consisting of data provided by the authors to benefit the reader, the posted materials are not copyedited and are the sole responsibility of the authors, so questions or comments should be addressed to the corresponding author.
