# Supplementary material for: Non-English Preferred Language and Hospital Mortality in Candidemia: A Retrospective Cohort Study
Source: Open Forum Infect Dis. 2026 Oct 8;13(10):ofag626. doi: 10.1093/ofid/ofag626 (PMC13660683; doi:10.1093/ofid/ofag626)
Supplement: Supplementary file 1 [file ofag626_supplementary_data.docx]

Supplementary Materials for: **Non-English Preferred Language and Hospital Mortality in Candidemia: A Retrospective Cohort Study**

**Supplemental Methods: Statistical Modeling**

*Primary model (Model A)*

Logistic regression evaluating non-English preferred language and hospital mortality, adjusted for age, sex, diabetes, ESRD, vasopressor use, mechanical ventilation, and Candida species.

*Secondary SDOH models (Models A2–A5)*

Separate models were constructed by replacing non-English preferred language with housing insecurity (A2), insurance category (A3), substance use history (A4), and partnered status (A5), while retaining the same clinical covariates.

*Race/ethnicity model (Model A-Race)*

Model A with additional adjustment for race (Black vs non-Black) and Latino ethnicity.

*Neighborhood model (Model AN)*

Model A with inclusion of national ADI percentile. The use of national ADI percentile as a measure of neighborhood disadvantage is an area-level proxy and may not accurately reflect individual social circumstances.

*Care-process model (Model B)*

Model A with additional adjustment for repeat blood cultures and ophthalmologic examination.

**Supplemental Table 1.** Sensitivity analyses evaluating the potential impact of recurrent candidemia episodes on the association between non-English preferred language and hospital mortality

| **Analysis** | **N** | **aOR** | **95% CI** | **p** |
| --- | --- | --- | --- | --- |
| Primary episode-level analysis | 237 | 3.22 | 1.03–10.08 | .042 |
| Episode-level analysis with patient-clustered robust SEs | 237 | 3.22 | 0.97–10.69 | .056 |
| First candidemia episode per patient | 199 | 3.18 | 1.01–10.11 | .046 |

All models were adjusted for age, sex, diabetes, end-stage renal disease, vasopressor use, mechanical ventilation, and Candida albicans species. The patient-clustered analysis used robust standard errors clustered by unique patient identifier.

**Supplemental Table** **2.** Secondary “swap-in” sensitivity model (A2): multivariable logistic regression for hospital mortality with housing insecurity as the primary SDOH exposure (N=202 episodes, 35 deleted due to missingness).

| **Predictor** | **aOR** | **95% CI** | **p** |
| --- | --- | --- | --- |
| Housing insecurity | 0.53 | 0.16–1.57 | 0.264 |
| Age (per year) | 1.04 | 1.02–1.07 | 0.002 |
| Male sex | 0.66 | 0.30–1.48 | 0.319 |
| Diabetes | 1.09 | 0.44–2.64 | 0.850 |
| ESRD | 0.71 | 0.08–4.77 | 0.734 |
| Vasopressor required | 4.54 | 1.48–14.16 | 0.008 |
| Mechanical ventilation | 1.52 | 0.49–4.74 | 0.466 |
| *Candida albicans* (vs non-*albicans*) | 0.41 | 0.16–0.99 | 0.054 |

**Supplemental Table 3.** Secondary “swap-in” sensitivity model (A3): multivariable logistic regression for hospital mortality with insurance category (private as reference) as the primary exposure (N=237).

| **Predictor** | **aOR** | **95% CI** | **p** |
| --- | --- | --- | --- |
| Dual coverage (vs Private) | 0.36 | 0.09–1.30 | 0.126 |
| Medicaid only (vs Private) | 0.63 | 0.20–1.99 | 0.426 |
| Medicare only (vs Private) | 0.48 | 0.15–1.54 | 0.218 |
| Uninsured/none (vs Private) | 4.20 | 0.66–24.01 | 0.109 |
| Age (per year) | 1.06 | 1.03–1.09 | <0.001 |
| Male sex | 0.64 | 0.30–1.37 | 0.251 |
| Diabetes | 0.85 | 0.37–1.92 | 0.697 |
| ESRD | 1.20 | 0.19–6.65 | 0.841 |
| Vasopressor required | 4.35 | 1.53–12.49 | 0.006 |
| Mechanical ventilation | 1.89 | 0.67–5.40 | 0.229 |
| *Candida albicans* (vs non-*albicans*) | 0.58 | 0.25–1.28 | 0.182 |

**Supplemental Table 4.** Secondary “swap-in” sensitivity model (A4): multivariable logistic regression for hospital mortality with history of substance use as the primary SDOH exposure (N=237)

| **Predictor** | **aOR** | **95% CI** | **p** |
| --- | --- | --- | --- |
| Any history of substance use | 0.45 | 0.16–1.16 | 0.110 |
| Age (per year) | 1.04 | 1.01–1.06 | 0.002 |
| Male sex | 0.78 | 0.38–1.61 | 0.500 |
| Diabetes | 0.88 | 0.39–1.96 | 0.764 |
| ESRD | 1.30 | 0.19–8.03 | 0.780 |
| Vasopressor required | 4.76 | 1.69–13.54 | 0.003 |
| Mechanical ventilation | 1.70 | 0.61–4.81 | 0.310 |
| *Candida albicans* (vs non-*albicans*) | 0.66 | 0.30–1.42 | 0.300 |

**Supplemental Table 5.** Secondary “swap-in” sensitivity model (A5): multivariable logistic regression for hospital mortality with partnered status as the primary exposure (N=237).

| **Predictor** | **aOR** | **95% CI** | **p** |
| --- | --- | --- | --- |
| Partnered marital status (yes vs no) | 0.90 | 0.40–2.00 | 0.796 |
| Age (per year) | 1.04 | 1.02–1.07 | <0.001 |
| Male sex | 0.72 | 0.35–1.49 | 0.378 |
| Diabetes | 0.85 | 0.37–1.87 | 0.681 |
| ESRD | 0.94 | 0.16–4.94 | 0.945 |
| Vasopressor required | 4.83 | 1.74–13.62 | 0.003 |
| Mechanical ventilation | 1.60 | 0.58–4.43 | 0.363 |
| *Candida albicans* (vs non-*albicans*) | 0.68 | 0.31–1.48 | 0.343 |

**Supplemental Table 6.** Race/ethnicity–adjusted sensitivity model (Model A-Race): multivariable logistic regression for hospital mortality among candidemia episodes.

| **Predictor** | **aOR** | **95% CI** | **p** |
| --- | --- | --- | --- |
| Non-English preferred language | 4.56 | 1.19–18.81 | 0.029 |
| Black race (vs non-Black) | 1.40 | 0.59–3.28 | 0.440 |
| Hispanic/Latino ethnicity (yes vs no) | 0.63 | 0.16–2.12 | 0.473 |
| Age (per year) | 1.04 | 1.02–1.07 | <0.001 |
| Male sex | 0.63 | 0.30–1.32 | 0.225 |
| Diabetes | 0.82 | 0.35–1.83 | 0.627 |
| ESRD | 0.61 | 0.09–3.62 | 0.596 |
| Vasopressor required | 4.95 | 1.72–14.43 | 0.003 |
| Mechanical ventilation | 1.62 | 0.57–4.67 | 0.363 |
| *Candida albicans* (vs non-*albicans*) | 0.75 | 0.33–1.63 | 0.471 |

**Supplemental Table 7.** Neighborhood-augmented sensitivity model (AN): multivariable logistic regression for hospital mortality including national ADI (N=227 episodes, 10 deleted due to missingness).

| **Predictor** | **aOR** | **95% CI** | **p** |
| --- | --- | --- | --- |
| Non-English preferred language | 3.98 | 1.22–13.39 | 0.022 |
| ADI national (per 1 unit) | 1.00 | 0.98–1.02 | 0.916 |
| Age (per year) | 1.04 | 1.02–1.07 | <0.001 |
| Male sex | 0.76 | 0.36–1.61 | 0.470 |
| Diabetes | 0.89 | 0.38–2.03 | 0.776 |
| ESRD | 0.17 | 0.007–1.88 | 0.190 |
| Vasopressor required | 5.00 | 1.69–15.11 | 0.004 |
| Mechanical ventilation | 1.71 | 0.58–5.10 | 0.328 |
| *Candida albicans* (vs non-*albicans*) | 0.72 | 0.32–1.58 | 0.418 |
